# Рекомбинантный укороченный белок TNF-BD вируса натуральной оспы проявляет специфическую фармакологическую активность в экспериментальной модели септического шока

**DOI:** 10.18699/VJ20.616

**Published:** 2020-05

**Authors:** И.П. Гилева, С.Н. Якубицкий, И.В. Колосова, С.Н. Щелкунов

**Affiliations:** Государственный научный центр вирусологии и биотехнологии «Вектор» Роспотребнадзора, р. п. Кольцово, Новосибирская область, Россия; Государственный научный центр вирусологии и биотехнологии «Вектор» Роспотребнадзора, р. п. Кольцово, Новосибирская область, Россия; Государственный научный центр вирусологии и биотехнологии «Вектор» Роспотребнадзора, р. п. Кольцово, Новосибирская область, Россия; Государственный научный центр вирусологии и биотехнологии «Вектор» Роспотребнадзора, р. п. Кольцово, Новосибирская область, Россия Федеральный исследовательский центр Институт цитологии и генетики Сибирского отделения Российской академии наук, Новосибирск, Россия

**Keywords:** VARV-CrmB protein, variola virus, LPS, septic shock, TNF-biding domain, белок VARV-CrmB, вирус натуральной оспы, ЛПС, септический шок, TNF-связывающий домен

## Abstract

Фактор некроза опухолей (TNF) – один из основных цитокинов, медиаторов иммунной системы, обеспечивающих защиту организма человека от вирусных инфекций. В процессе эволюции антропогенный вирус натуральной оспы (Variola virus, VARV) освоил эффективные механизмы преодоления иммунологических барьеров человека, кодируя в своем геноме белки, способные взаимодействовать с рецепторами цитокинов организма-хозяина, блокируя таким образом их активность. В частности, ген G2R этого вируса кодирует белок CrmB, который эффективно связывает TNF человека и мышей. При этом данный белок является двухдоменным и наряду с TNF-связывающим N-концевым доменом содержит С-концевой хемокин-связывающий домен. При использовании методологии молекулярного клонирования нами ранее получен рекомбинантный бакуловирус, продуцирующий в клетках насекомых рекомбинантный белок CrmB вируса натуральной оспы (VARV-CrmB), и показан его TNF-нейтрализующий потенциал в различных тестах in vitro и in vivo. С целью снижения иммуногенности этого вирусного белка при его многократном введении для терапии хронических воспалительных заболеваний получена рекомбинантная плазмида, направляющая в клетках Escherichia coli биосинтез укороченного однодоменного TNF-связывающего (TNF-BD) белка VARV. Методом металл-хелатной аффинной хроматографии из клеток выделен рекомбинантный белок TNF-BD. Терапевтический потенциал белка TNF-BD изучен в экспериментальной модели септического шока, индуцированного в организме мышей введением бактериального липополисахарида (ЛПС). После индукции септического шока животным вводили разные дозы рекомбинантного белка TNF-BD и регистрировали их гибель в течение 7 сут. Все мыши, не получавшие препарат белка TNF-BD после инъекции ЛПС, погибли через 3 сут, а в группах животных, которым вводили TNF-BD, в зависимости от дозы этого белка выжили 30, 40 или 60 % мышей. Результаты исследования демонстрируют наличие специфической фармакологической активности у рекомбинантного белка TNF-BD, синтезированного в бактериальных клетках, в мышиной модели ЛПС-индуцированного септического шока.

## Abstract (en)

Tumor necrosis factor (TNF) is one among the key cytokines that mediate the immune system to protect humans against viral infections. Throughout evolution, anthropogenic Variola virus (VARV) has developed effective mechanisms to overcome human defense reactions. The viral genome encodes soluble proteins imitating the structure of cellular cytokine receptors. These proteins compete with cellular receptors for cytokine binding, thus blocking the antiviral immune response. In particular, the G2R gene of VARV encodes the TNF decoy receptor, VARV-CrmB protein. This protein consists of N-ended TNF-biding (TNF-BD) and C-ended chemokine binding (Ch-BD) domains. Recombinant VARV-CrmB protein has been produced in insect cells using molecular cloning methods and its TNF neutralizing activity has been shown in vitro and in vivo. To decrease the immunogenicity of this protein, a recombinant plasmid coding for shortened TNF-BD protein of VARV in Escherichia coli cells has been constructed. Using the method of immobilized metal affinity chromatography, recombinant TNF-BD protein corresponding to the TNF-biding domain of VARV-CrmB protein was purified from E. coli cells. The therapeutic potential of TNF-BD was studied using an experimental model of LPS-induced septic shock. After septic shock induction, several doses of recombinant TNF-BD were injected and the mortality of experimental animals was observed during 7 days. All mice not injected with TNF-BD had been dead by day 3 of the experiment, but 30, 40 and 60 % of the experimental animals, who received different TNF-BD doses, survived in a dose-dependent manner. Data obtained demonstrate that recombinant TNF-BD protein is pharmacologically active in the experimental model of LPS-induced septic shock.

## Введение

В процессе коэволюции у вируса натуральной оспы (ВНО) появились многочисленные эффективные механизмы преодоления иммунологических барьеров человека. В частности, вирусный геном детерминирует синтез секретируемых белков, имеющих структурное сходство с клеточными рецепторами цитокинов. Вирусные белки функционируют как связывающие цитокины рецепторы, блокируя таким образом их активность (Shchelkunova, Shchelkunov, 2016). Способность вирусных белков эффективно взаимодействовать с иммунной системой человека открывает перспективу их использования для разработки иммуномодулирующих терапевтических средств нового поколения.

Некоторые TNF-блокаторы – Remicade® (Centocor, Inc., Malvern, PA, США), Enbrel® (Immunex Corp, Seattle, WA, США), Humira™ (Abbott Laboratories, Abbott Park, IL, США), действующим началом которых являются моно- клональные антитела или химерные белки, состоящие из TNF-рецепторных и иммуноглобулиновых доменов, прошли клинические испытания и разрешены для применения в медицинской практике, но не один из перечисленных препаратов не оказался эффективным против септического шока (Monaco et al., 2015). Продемонстрированная in vivo и in vitro высокая TNF-нейтрализующая активность белка VARV-CrmB вируса натуральной оспы (Gileva et al., 2006; Shchelkunova, Shchelkunov, 2016) позволяет надеяться, что основой терапевтических пре- паратов нового поколения может стать этот белок.

Эндотоксический шок, индуцированный липополисахаридом (ЛПС) грамотрицательных бактерий (реакция Шварцмана), – широко используемая экспериментальная модель септического шока (Leturcq et al., 1995; Lamping et al., 1998; Fei et al., 2011). Введение рекомбинантного белка VARV-CrmB мышам с ЛПС-индуцированным септическим шоком приводило к достоверному увеличению выживаемости мышей и отчетливому снижению гистопатологических изменений внутренних органов по срав- нению с контрольными животными (Gileva et al., 2006).

Полноразмерный рекомбинантный белок VARV-CrmB имеет молекулярную массу 47 кДа, и в его структуре выделяют N-концевой TNF- (TNF-BD) и С-концевой хемокин-связывающий (Сh-BD) домены (Alejo et al., 2006). С целью уменьшения иммуногенности рекомбинантного белка VARV-CrmB ранее нами получен его укороченный однодоменный вариант TNF-BD. Показаны биологическая активность белка TNF-BD in vitro (ингибирование как цитотоксичности TNF для культуры клеток фибробластов мыши L929, так и TNF-индуцированной окислительно- метаболической активности лейкоцитов крови мышей) (Цырендоржиев и др., 2014; Трегубчак и др., 2015), а также пониженная иммуногенность относительно полнораз- мерного белка (Непомнящих и др., 2017).

Цель настоящего исследования – изучение специфи- ческой фармакологической активности укороченного однодоменного рекомбинантного белка TNF-BD вируса натуральной оспы in vivo, а именно – в экспериментальной модели ЛПС-индуцированного септического шока.

## Материалы и методы

Реагенты. Для приготовления жидких и твердых пита- тельных средств использовали бактопептон, дрожжевой экстракт и бактоагар (Difco, США), антибиотики ампициллин, тетрациклин и канамицин (Sigma, США). Ре- комбинантную плазмидную ДНК выделяли с помощью набора Qiagen Plasmid Midi Kit (QIAGEN, Германия). В работе были применены также реактивы фирмы Sigma (США): имидазол, ЛПС Escherichia coli 055:B5, мочевина, фенилметилсульфонилфторид (PMSF), IPTG.

Рекомбинантные плазмиды и бактериальные штаммы. Рекомбинантная плазмида pQE60-TNF-BD любезно предоставлена Т.В. Трегубчак (Трегубчак и др., 2015). Для амплификации этой плазмиды использовали штамм E. coli XL10-Gold (QIAGEN, Германия), а для нара- ботки рекомбинантного белка TNF-BD – штамм E. coli SG13009[pRep4] (QIAGEN, Германия). Компетентные клетки E. coli XL10-Gold и E. coli SG13009 получали по методу (Mandel, Higa, 1970).

Бактериальные среды. Для культивирования штамма E. coli SG13009[pRep4], содержащего pQE60-TNF-BD, ис- пользовали стандартную LB-среду (Sambrook et al., 1985).

Электрофорез, хроматография. Фракционирование белков проводили в SDS-ПААГ по стандартной методике (Laemmli, 1970), с помощью маркеров молекулярных масс белков PageRuler™ Plus Prestained Protein Ladder (Thermo Scientific, США) (11, 17, 28, 36, 55, 72, 95, 130, 250 кДа). Металл-хелатную афинную хроматографию проводили, используя Ni-NTA агарозу (QIAGEN, Германия), на ко- лонке объемом 1 мл. Концентрацию белка определяли по методу (Bradford, 1976).

Животные. В эксперименты брали самок мышей линии BALB/c с массой тела 19–21 г (возраст 8–9 нед), полученных из вивария Государственного научного центра вирусологии и биотехнологии «Вектор» Роспо- требнадзора (Кольцово, Новосибирская область, Россия). Мышей содержали при естественном световом режиме и постоянном доступе к воде и пище. Животных выводили из эксперимента в соответствии с правилами, принятыми Европейской конвенцией по защите животных, используемых для экспериментальных целей.

## Результаты

Наработка рекомбинантного белка TNF-BD. Для полу- чения инокулятов были взяты индивидуальные колонии Ap^r^-, Km^r^-, TcM^r^-трансформантов E. coli SG13009[pRep4]. Инокулят разводили свежим L-бульоном (1:50), содержа- щим 100 мкг/мл ампициллина и по 30 мкг/мл канамицина и тетрациклина. Бактериальную культуру инкубировали при 37 °С и интенсивной аэрации до достижения оптической плотности культуры А_550_ = 0.3–0.5, затем добавляли индуктор IPTG до концентрации 1 мМ (продолжительность индукции 4 ч). Клетки собирали низкоскоростным центрифугированием и ресуспендировали в 5 мл буфера следующего состава: 10 % сахароза, 100 мМ Трис-HCl, pH 7.3, 5 мМ ЕДТА, 20 мкг/мл PMSF. Затем бактериальные клетки разрушали ультразвуком на установке Soniprep (MSE, США; 10 циклов по 1 мин с интервалами между циклами в 30 с). Как было показано ранее (Трегубчак и др., 2015), рекомбинантный белок TNF-BD синтезируется в клетках E. coli в виде «телец включения». Для выделения целевого продукта тельца включения солюбилизовали в буфере А (100 мМ NaH_2_PO_4_, 10 мМ Трис-НCl, pH 8.0, 300 мМ NaCl, 10 мМ имидазол, 6 М мочевина) и суперна- тант наносили на колонку с Ni-NTA агарозой (V = 1 мл), уравновешенную 5 мл этого же буфера. Колонку про- мывали (2 мл × 3) буфером В (100 мМ NaH2PO4, 10 мМ Трис-НCl, pH 6.3, 300 мМ NaCl, 20 мМ имидазол, 6 М мочевина) и элюировали целевой продукт буфером С (100 мМ NaH_2_PO_4_, 10 мМ Трис-НCl, pH 8.0, 300 мМ NaCl, 250 мМ имидазол) (0.3 мл × 5). Фракции выделения анализировали в 10 % ПААГ. Результаты выделения представлены на рис. 1. Фракции, содержащие целевой продукт, объединяли. Таким образом, был выделен пре- парат рекомбинантного белка TNF-BD, чистота которого, по данным электрофореза, составляла около 90 %.

Исследование токсичности рекомбинантного белка TNF-BD. Для того чтобы определить способность пре- парата укороченного однодоменного белка TNF-BD предотвращать взаимодействие мышиного TNF со специфи- ческими рецепторами эукариотических клеток, изучали его активность в тесте нейтрализации цитотоксического действия mTNF на культуре клеток мышиных фибробластов L929, как описано в работе (Трегубчак и др., 2015). Для определения токсичности рекомбинантного белка взяли две группы мышей линии BALB/c (каждая группа содержала пять животных). Одной группе вводили внутрибрюшинно физиологический раствор, другой – ре- комбинантный белок в дозе 50 мкг/мышь. Наблюдение вели в течение 7 сут, за этот период не было обнаружено проявлений токсичности рекомбинантного белка.

Исследование специфической фармакологической активности белка TNF-BD в экспериментальной модели септического шока. Предварительно для мышей линии BALB/c была определена доза ЛПС E. coli 055:B5, вызывающая гибель 100 % животных (ЛД100 ), равная 20 мг/ кг веса. Животные были объединены в пять групп (по 10 особей в группе). Мышам в группах 1–3 внутрибрюшинно вводили рекомбинантный белок TNF-BD в дозах 0.2, 2 или 20 мкг/мышь. Через 30 мин этим живот- ным внутрибрюшинно инъецировали раствор ЛПС E. coli (O55:B5) в дозе 1 ЛД100. Группе сравнения (группа 4) вводили только ЛПС, а группе отрицательного контроля (группа 5) – натрий-фосфатный буфер. После введения препаратов мышам регистрировали их гибель в течение 7 сут. Результаты эксперимента показаны на рис. 2: на конец эксперимента выживаемость животных в группе 5 составила 100 %, в группе 4 – 0 %, а в группах 1–3 – 30, 40 и 60 % соответственно.

**Fig. 1.**
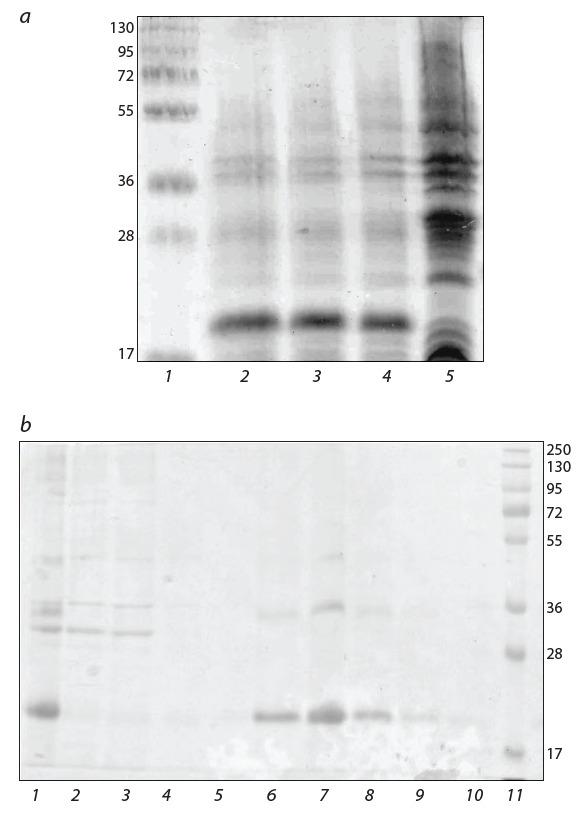
Electrophoretic resolution of proteins. (a) PAGE resolution of E. coli cell lysates with 10 % SDS. Lanes: 1, protein ladder; 2–4, SG13009[pRep4, pQE60-TNF-BD]; 5, SG13009[pRep4]. (b) PAGE resolution of recombinant TNF-BD protein fractions with 12 % SDS. Lanes: 1, fraction of insoluble cell proteins of E. coli SG13009[pRep4, pQE60-TNF-BD] solubilized in buffer A; 2, 3, loading onto a column with Ni-NTA agarose equilibrated with buffer A; 4, 5, washing the column with buffer B; 6–10, elution of the target product with buffer C; 11, protein ladder.

**Fig. 2.**
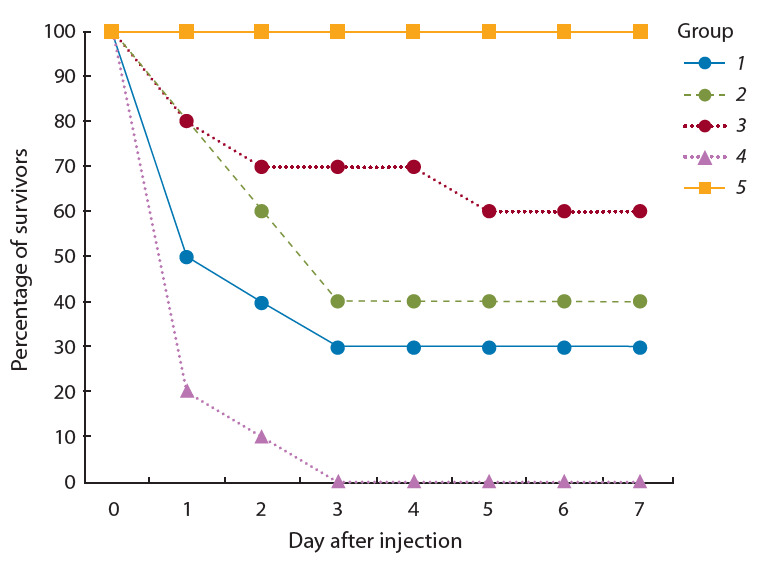
Survival of experimental animals with LPS-induced endotoxic shock after administration of the recombinant TNF-BD protein.

## Обсуждение

Cигнальная система, активируемая TNF, – один из основных факторов, обусловливающих развитие воспалительного процесса. Гиперпродукция TNF может приводить к гибели организма в результате развития системной воспалительной реакции или септического шока. Сепсис и септический шок до настоящего времени представляют одну из основных проблем здравоохранения. Ежегодно они являются причиной смерти более миллиона человек (Seymour, Rosengart, 2015). TNF-антагонисты, действующим началом которых были моноклональные антитела (Infliximab, Adalimumab) или аналог клеточных рецепторов (Etanercept), прошли клинические испытания и разрешены для применения в медицинской практике, но не один из этих препаратов не проявил терапевтической активности против септического шока (Monaco et al., 2015). Поэтому поиск новых классов соединений, обладающих TNF-нейтрализующей активностью, остается актуальной проблемой.

Существование вирусных белков, способных связывать цитокины и, в частности TNF, блокируя таким образом его активность (Shchelkunova, Shchelkunov, 2016), позволяет предположить возможность разработки нового поколения TNF-антагонистов на основе TNF-связывающего белка вируса натуральной оспы (VARV-CrmB). Ранее нами по- лучен рекомбинантный бакуловирус BVi67, несущий ген белка VARV-CrmB, а из инфицированных им клеток насекомых линии Sf21 выделен рекомбинантный вирусный белок. Рекомбинантный белок VARV-CrmB эффективно нейтрализует эффекты TNF в экспериментальных системах in vitro и in vivo (Gileva et al., 2006). Особенно следует подчеркнуть его выраженный терапевтический эффект на проявление ЛПС-индуцированного септического шока у мышей, что отчетливо снижает гистопатологические из- менения внутренних органов и достоверно увеличивает выживаемость животных (Gileva et al., 2006). Новый TNF-антагонист, кроме TNF-нейтрализующего потенциала, должен обладать низкой иммуногенностью, так как эффективность применения препарата может быть снижена из-за развития иммунного ответа на терапевтический белок (Chen et al., 2015; Eng et al., 2015).

Полноразмерный рекомбинантный белок VARV-CrmB (47 кДа) обладает способностью к олигомеризации, это может снизить его терапевтический потенциал при по- вторном или многократном применении. Белок VARVCrmB состоит из TNF- и хемокин-связывающего доменов (Alejo et al., 2006). Нами сделано предположение, что однодоменный укороченный TNF-связывающий белок сохранит свою терапевтическую активность и будет обладать существенно меньшей иммуногенностью. Был получен бактериальный продуцент укороченного белка TNF-BD, соответствующего TNF-связывающему домену белка VARV-CrmB, и экспериментально доказана его способность ингибировать цитотоксичность TNF на клетках мышиных фибробластов L929 и TNF-индуцированную окислительно-метаболическую активность лейкоцитов крови мышей (Цырендоржиев и др., 2014; Трегубчак и др., 2015). При этом рекомбинантный TNF-BD проявлял сниженную иммуногенность относительно белка VARVCrmB при многократном введении экспериментальным животным (Непомнящих и др., 2017).

В настоящем исследовании изучен терапевтический потенциал короткого рекомбинантного однодоменного белка TNF-BD в экспериментальной модели септического шока. С этой целью белок TNF-BD выделили из клеток E. coli методом металл-хелатной аффинной хроматографии, как это показано на рис. 1, а и б. При его внутрибрюшинном введении мышам линии BALB/c установлено отсутствие токсичности. Для мышей этой линии экспериментально определена доза ЛПС E. coli (O55:B5), вызывающая 100 % гибель животных (ЛД100 ). При внутрибрюшинном введении ЛПС и белка TNF-BD в нашей работе впервые продемонстрирована дозозависимая специфическая фармакологическая активность низкоиммуногенного укороченного рекомбинантного белка TNF-BD в модели ЛПС- индуцированного септического шока (см. рис. 2).

Следует отметить, что вопрос правомерности использования мышиной модели эндотоксимии (реакции Щварцмана) для выявления новых препаратов для терапии септического шока у людей остается дискуссионным. Основанием для скептического отношения к мышиной модели служат отличия результатов транскриптомных анализов, механизмов развития врожденного и приобретенного иммунных ответов, гетерогенность человеческой популяции versus гомогенности инбредных линий мышей (Efron et al., 2015; Stortz et al., 2017). Не исключено, что препараты, проявившие высокую эффективность в мыши- ной модели сепсиса, не будут эффективными для человека, как это случилось с препаратом Centocor (Thayer, 1993). Однако простота проведения эксперимента и воспроизводимость результатов делают эту модель незаменимой для первичного скрининга TNF-связывающих терапевтических препаратов.

## Заключение

На основании проведенного исследования можно сделать вывод, что рекомбинантный белок TNF-BD вируса нату- ральной оспы является перспективным для разработки нового поколения TNF-антагонистов. Этот белок может служить модельным объектом для изучения взаимо- действия вирусных белков с компонентами иммунной системы организма человека. Методами делеционного анализа и мутагенеза, рентгеноструктурного анализа, компьютерного моделирования возможно идентифициро- вать в структуре белка TNF-BD аминокислотные остатки, осуществляющие взаимодействие с TNF и составляющие конкуренцию клеточным рецепторам.

## Conflict of interest

The authors declare no conflict of interest.
